# The Value of Surgery for Retroperitoneal Sarcoma

**DOI:** 10.1155/2009/605840

**Published:** 2009-10-08

**Authors:** Sepideh Gholami, Charlotte D. Jacobs, Daniel S. Kapp, Layla M. Parast, Jeffrey A. Norton

**Affiliations:** ^1^Stanford University School of Medicine, Stanford, CA 94305, USA; ^2^Department of Medicine, Stanford University School of Medicine, Stanford, CA 94305, USA; ^3^Department of Radiation Oncology, Stanford University School of Medicine, Stanford, CA 94305, USA; ^4^Department of Biostatistics, Harvard School of Public Health, Boston, MA 02115, USA; ^5^Department of Surgery, Stanford University, Stanford, CA 94305, USA

## Abstract

*Introduction*. Retroperitoneal sarcomas are uncommon large malignant tumors. *Methods*. Forty-one consecutive patients with localized retroperitoneal sarcoma were retrospectively studied. *Results*. Median age was 58 years (range 20–91 years). Median tumor size was 17.5 cm (range 4–41 cm). Only 2 tumors were <5 cm. Most were liposarcoma (44%) and high-grade (59%). 59% were stage 3 and the rest was stage 1. Median followup was 10 months (range 1–106 months). Thirty-eight patients had an initial complete resection; 15 (37%) developed recurrent sarcoma and 12 (80%) had a second complete resection. Patients with an initial complete resection had a 5-year survival of 46%. For all patients, tumor grade affected overall survival (*P* = .006). Complete surgical resection improved overall survival for high-grade tumors (*P* = .03). *Conclusions*. Tumor grade/stage and complete surgical resection for high-grade tumors are important prognostic variables. Radiation therapy or chemotherapy had no significant impact on overall or recurrence-free survival. Complete surgical resection is the treatment of choice for patients with initial and locally recurrent retroperitoneal sarcoma.

## 1. Introduction

Sarcomas are uncommon malignant tumors arising from mesenchymal tissue. Retroperitoneal sarcomas account for approximately 10% of soft tissue sarcomas and less than 1% of all malignant neoplasms [1, 2]. The common histologic types are liposarcoma, leiomyosarcoma, and malignant fibrous histiocytoma [3–6].

Due to minimal early symptoms, retroperitoneal sarcomas are often diagnosed when the tumors are large and involve surrounding organs. En bloc surgical resection is the treatment of choice. This has been described recently as compartmental resection in which there is a systematic removal of adjacent organs to obtain a rim of normal tissue surrounding the tumor [7]. Compartmental resection has been reported to have a 3-fold lower rate of sarcoma recurrence in a recent study [7]. In general, surgery has a reported complete resection rate between 65–99% [4, 5]. However, complete resection may be difficult to achieve, due to involvement of surrounding vital structures. The completeness of surgical resection is an important prognostic variable as it has been shown to improve survival [7–9]. Incomplete resection is ineffective [5, 8].

In contrast to patients with extremity sarcomas, the efficacy of adjuvant radiation therapy for retroperitoneal sarcomas is less clear. One of the major limiting factors is difficulty in delivering sufficient radiation dose because of toxicity to adjacent organs including bowel, kidneys, liver, and spinal cord [10, 11]. Two randomized trials have demonstrated that adjuvant radiation therapy results in a significant reduction in local recurrence rates in superficial trunk and extremity sarcomas [11, 12]. One randomized trial using intraoperative radiation has also shown an improvement in local control of retroperitoneal sarcomas [10]. In addition, several retrospective and prospective studies suggest improved local control with the use of adjuvant radiation therapy for retroperitoneal sarcomas [13–15]. Catton et al. showed that postoperative radiation therapy with doses of >35 Gy after complete resection of the tumor delayed, although did not prevent, local recurrence when compared to historical controls [13]. Fein et al. demonstrated a lower local failure rate in patients with retroperitoneal sarcoma who received adjuvant radiation therapy [16]. However, a recent study by Ballo et al. failed to show any improvement in local control with external beam or intra-operative radiation therapy [3]. Given the high relapse rate, some have offered patients with retroperitoneal sarcoma adjuvant chemotherapy; however, its role remains unproven and controversial. The purpose of the current study was to review our experience in the management of retroperitoneal soft tissue sarcomas and identify important prognostic factors for local control and survival.

## 2. Materials and Methods

### 2.1. Patient Material

Between January 1996 and 2007, 102 consecutive patients with a presumptive diagnosis of retroperitoneal sarcoma were referred to Stanford Hospital and Clinics. Among these, eight patients were found to have gastrointestinal stromal tumors, one a desmoplastic round small cell tumor, and ten non-cancerous disease. Thirty-four patients had a previous surgical resection at another institution, and two had medical contraindications to major surgery. Six patients had distant metastatic disease. The remaining forty one patients had previously untreated localized retroperitoneal sarcoma and comprise the cohort of this study. Medical records, including clinic notes, radiographic, operative, and pathology reports were reviewed. The association between patient demographics, tumor characteristics, treatment variables, and outcomes was assessed. Patient demographics included sex, age, and duration of symptoms. Tumors were characterized based on histopathologic type, size in greatest dimension, grade, and pathological stage. We used the sixth edition of the American Joint Committee on Cancer (AJCC) staging system for soft tissue sarcomas. It categorizes stages 1–4 based on tumor size, depth, grade, lymph node, and distant metastases [17]. Treatment variables analyzed included complete (R0/R1) versus incomplete resection (R2) and adjuvant therapy. The use of radiation therapy and chemotherapy, recommended by a multidisciplinary team, was also assessed. This study was approved by the Human and Subjects Research Committee at Stanford University School of Medicine.

### 2.2. Statistical Analysis

Statistical analysis was performed with SAS software (version 9.1.3 sp4, SAS institution). Overall and disease-free survival was calculated. Date of diagnosis was defined as the time of initial tissue diagnosis. Local recurrence was defined as biopsy proof of tumor recurrence at the primary tumor site. Distant recurrence was defined as recurrent tumor at a site distant from the primary tumor. Overall survival was calculated from time of diagnosis to time of last followup or death. Deaths were confirmed by an internet-based search via the Social Security Death Index (http://ssdi.rootsweb.com). First recurrence was used as the endpoint for disease-free survival. Kaplan-Meier method and the log-rank test were utilized for univariate analysis and comparison of studied variables. The Cox-regression proportional hazards model was used for multivariate analysis of prognostic factors significant in the univariate analysis. All *P*-values recorded are two-sided and a *P*-value <.05 was considered statistically significant.

## 3. Results

### 3.1. Patient and Tumor Characteristics

Table 1 summarizes the demographic characteristics of 41 patients with primary retroperitoneal sarcoma included in this study. Twenty-three (56%) were men and 18 (44%) woman. Median age was 58 years (range 20–91 years). Twenty-five patients (61%) reported symptoms of less than 6 months duration and 11 (27%) had symptoms for longer periods of time. Five patients did not have a record of symptoms. Presenting symptoms included pain (*n* = 21.51%), mass/abdominal distention (*n* = 12.29%), constitutional symptoms (*n* = 3), and others (*n* = 5). Liposarcoma was the most common histological type (54%), followed by leiomyosarcoma (17%). Median tumor size was 17.5 cm (range 4–41 cm); 59% of the sarcomas were high grade. Seventeen patients (41%) had stage 1 (low-grade sarcomas, <5 cm without metastases), and 24 (59%) had stage 3 (high-grade sarcomas, >5 cm without metastases) disease. Median followup was 10 months (range 1–106 months).

### 3.2. Treatment

#### 3.2.1. Surgery

All patients underwent abdominal retroperitoneal exploration using an open approach. A total of 67 surgical procedures were performed in 41 patients; 18 patients underwent multiple surgical procedures. At time of resection, 38 patients (93%) had a complete resection with negative macroscopic margins (R0/R1) (Table 2). The magnitude of surgery was significant because we tried to remove normal structures that were adjacent to the tumor. Sixty-eight percent had one or more contiguous organs removed to achieve a complete resection (kidney *n* = 18, colon *n* = 14, small intestine *n* = 6, spleen *n* = 4, pancreas *n* = 4, rectum/anus *n* = 3, liver *n* = 2, bladder *n* = 1, stomach *n* = 1, prostate *n* = 1, gallbladder *n* = 1). Morbidity rate was 19%. Complications included infection (*n* = 5), anastomotic leak (*n* = 2), post-operative hemorrhage (*n* = 2), small bowel obstruction (*n* = 2), and myocardial infarction (*n* = 2). Mortality rate was 2%.

Tumor recurred in fifteen patients (37%), with recurrent site local (*n* = 10), distant (*n* = 2), and both local and distant (*n* = 3). Of these, 12 (80%) underwent re-exploration and a second complete resection was achieved in all patients (100%). Ninety-three percent of patients had complete resection of the retroperitoneal sarcoma. Rates of complete resection were 100%, 86%, and 100% for the 1st, 2nd, and 3rd recurrences, respectively. CT imaging was able to accurately predict those who could have all tumor removed.

#### 3.2.2. Adjuvant Therapy

Nineteen (46%) patients had radiation therapy with their initial surgery, 10 (83%) with their second or third surgical resection. All patients with either R1 or R2 resection received adjuvant radiation therapy, whereas patients with R0 resections did not.

Radiation therapy with the initial surgical resection included either preoperative radiation therapy (*n* = 1), intra-operative or postoperative radiation therapy (*n* = 15), or a combination of both (*n* = 3). Post-operative external beam radiation (*n* = 6), intraoperative radiation (IORT) (*n* = 8), or both treatment modalities (*n* = 5) were administered. IORT dose was commonly 12.5 Gy and external beam radiation dose ranged from 45–50 Gy [17]. Eight patients (20%) also received neoadjuvant or adjuvant chemotherapy, including doxorubicin, ifosfamide, and mesna.

### 3.3. Overall Survival

**Figure 1:**
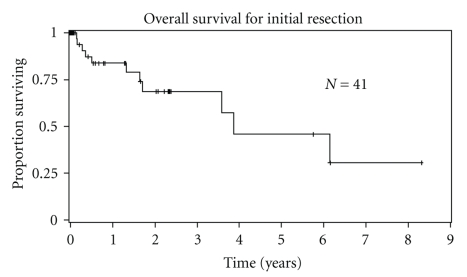
Overall survival of 41 patients with initial resection of primary retroperitoneal sarcoma.

**Figure 2:**
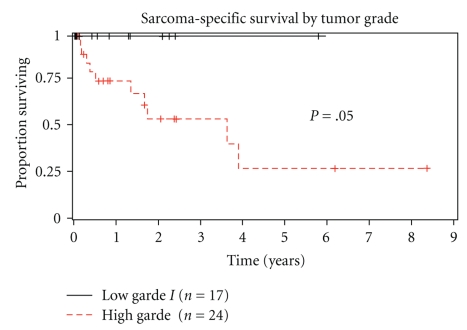
Sarcoma-specific survival of same 41 patients divided by tumor grade (low grade versus high grade).

Median overall survival for the 41 patients was 3.9 years. Estimated 2-year and 5-year survival was 69% and 46%, respectively (Figure 1). Gender, age, length of symptoms, histological type, and size of primary tumor did not significantly influence overall survival (Table 3). Tumor grade significantly impacted overall survival (Figure 2). Overall median survival for low-grade tumors was 6.1 years compared to 3.6 years for high-grade tumors (*P* = .05). Estimated 5-year survival for low-grade tumors versus high-grade tumors was 100% and 26%, respectively (Figure 2). When we included low-grade sarcomas, complete surgical resection of tumor did not improve overall survival. However, in a subset analysis including patients with only high-grade sarcomas, complete tumor resection improved overall survival. Adjuvant radiation or chemotherapy with initial surgery resection had no effect on overall survival. By multivariate analysis, only tumor grade and complete sarcoma resection for high-grade sarcomas affected overall survival.

### 3.4. Recurrence-Free Survival

**Figure 3:**
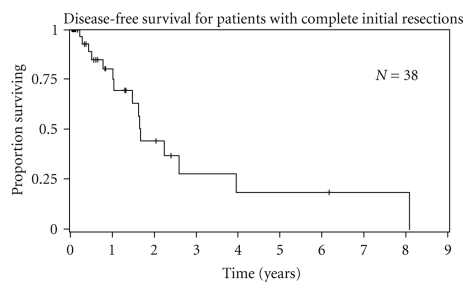
Disease-free survival of 38 patients who underwent complete initial resection of the primary retroperitoneal tumor.

4 (10%) patients developed distant metastases during subsequent followup such that most tumor recurrences were local. For patients with an initial complete resection (*n* = 38), median local recurrence-free survival was 1.65 years. Estimated 2-year and 5-year recurrence-free survival for patients with complete resections was 44% and 18%, respectively (Figure 3). Duration of symptoms had a significant impact on recurrence-free survival (Table 4). Patients reporting >6 months duration of symptoms at time of presentation had a shorter recurrence-free survival compared to patients with <6 months of symptoms (0.99 versus 3.9 years, *P* = .02). Adjuvant chemotherapy and radiation therapy did not significantly affect recurrence-free survival.

## 4. Discussion

Patients with retroperitoneal sarcomas tend to be diagnosed late when they have a very large tumor burden. This is the first study that demonstrates that duration of symptoms is inversely proportional to prognosis. This means that patients who have symptoms for longer periods of time without a diagnosis do worse and have a shorter recurrence-free survival. As other studies have shown, gross total surgical resection is the mainstay of treatment for retroperitoneal sarcoma [5, 7]. As expected, low-grade tumors have a better prognosis than high-grade tumors. For high-grade tumors complete resection did improve survival compared to incomplete resection. Our plan was not to simply remove the tumor. Instead, we removed adjacent organs rather than peel the tumor off. The pathology report seldom demonstrated tumor invasion of the proximate organ, but rather sarcoma abutment and encroachment. This approach is most similar to the recent report of complete compartmental resection with avoidance of tumor spilling [7]. However, complete surgical resection is often technically challenging and limited by invasion of adjacent nerves, blood vessels, and organs. Of the 41 patients with primary retroperitoneal tumors, an initial complete resection rate of 93% was achieved. To attain a complete resection, contiguous organs were removed in 68%, resulting in an estimated 5-year overall survival of 46%. This is comparable to the recent report in which the 5-year survival for high-grade sarcomas was 50% following complete resection [7]. This is comparable to other reported series with results ranging from 35–63% [12, 18] in which complete surgical resection was associated with improved survival.

Reported prognostic factors for overall survival for retroperitoneal sarcoma include grade, stage, histology, size, and margin status [4, 5, 14, 19, 20]. Due to the absence of early symptoms, failure to diagnose small retroperitoneal sarcomas remains a major issue. In our study, duration of symptoms influenced disease-free survival by univariate analysis. We also found that grade and complete resection for high-grade tumors were associated with overall survival by univariate (*P* < .05 and *P* < .03) as well as multivariate analysis (*P* < .006). This was also reported by Lewis et al., who showed that patients with low-grade tumors had a median survival of 149 months compared to only 33 months for high-grade tumors [5]. Therefore, the treatment goal for patients with initial presentation of retroperitoneal sarcoma should be complete surgical resection, even if adjacent normal organs need to be removed [5, 7, 12, 21].

A major challenge in treating retroperitoneal sarcomas today remains the high rate of local recurrence. Even when a complete resection has been achieved, local recurrence is the main site of treatment failure. For patients in our series who had a complete resection, the 5-year disease-free survival rate was only 18%. This is a discouraging result and one that needs further work. Local recurrences, even on multiple occasions, are still able to be treated by subsequent surgical resection. We followed our patients closely with imaging (CT +/or MRI) and local recurrences were identified early. This early recognition of recurrence allowed tumor to be completely removed surgically. Fortunately, we did not see sarcomatosis or multifocality as a pattern of recurrence that has been described by others as a potential result of tumor spill during the initial resection. Sarcomatosis has a much poorer prognosis [22]. For all patients with local recurrences a second complete resection was achieved 100% of the time. This is unusual because only one other series has reported resection rates for recurrences comparable to those for initial surgery [9]. Thus, 5-year overall survival was still 46% despite the fact that 22 patients developed recurrent tumor. Subsequent surgery was able to return them to disease-free status and allow survival.

The use of adjuvant therapy to reduce the probability of recurrence and distant metastases has long been a topic of dispute. In our series, we were unable to show that radiation therapy or chemotherapy had any significant impact on overall or recurrence-free survival. Other studies have suggested that adjuvant radiation therapy may decrease the probability of local recurrence [7, 9, 13, 14]. Newer radiation techniques including intensity modulated radiation therapy, respiratory guided therapy, image guided radiation therapy, proton or heavy ion radiation therapy, and stereotactic radiation therapy should permit a higher dose of radiation to be given with less normal tissue toxicity. A clinical trial of preoperative radiation for retroperitoneal sarcomas has been completed, and results will hopefully indicate benefit for this population of patients [23]. The benefits of chemotherapy for retroperitoneal sarcomas are even less clear. In our series, chemotherapy was beneficial in only a small number of patients. Although randomized trials suggest benefit from adjuvant chemotherapy for extremity sarcomas, no trial shows a clear improvement in outcome for retroperitoneal sarcomas [24, 25].

## 5. Conclusions

In conclusion, our results show that sarcoma tumor grade and complete resection are important prognostic variables in patients with primary retroperitoneal sarcomas. Aggressive complete surgical resection can be done safely in the vast majority of patients, and, in general is the only effective therapy. It remains the treatment of choice and for patients with initial and recurrent retroperitoneal sarcoma. The role of adjuvant radiation and chemotherapy needs to be for evaluated in multicenter randomized trials. We therefore recommend that surgery is to be optimized in the care of patients with retroperitoneal sarcomas. Advancements in tumor biology and selective targeted therapy should hopefully result in improved management of retroperitoneal sarcomas.

## Figures and Tables

**Table 1:** Demographic and tumor characteristics of 41 patients with primary retroperitoneal sarcoma who underwent surgical resection at Stanford Hospital from 1996 to 2007.

Variable	*N*	% of total
Sex		
* *Female	18	44
* *Male	23	56
Age		
* * <55 years	16	39
* * >55 years	25	61
Duration of symptoms		
* * <6 months	25	61
* * >6 months	11	27
* *Unkown	5	12
Histological subtype		
* *Liposarcoma	22	54
* *Leiomyosarcoma	7	17
* *Malignant fibrous histiocytoma	4	10
* *Other	8	19
Tumor size (maximum diameter)		
* * <5 cm	2	5
* *5–10 cm	4	10
* *10–20 cm	18	44
* * >20 cm	17	41
Tumor grade		
* *Low	17	41
* *High	24	59

**Table 2:** Initial treatment characteristics of 41 patients with primary retroperitoneal sarcoma who underwent surgical resection at Stanford Hospital from 1996 to 2007.

Variable	*N*	% of total
Resection		
* *R0/R1	38	93
* *R2	3	7
Contiguous organs resected		
* *Yes	28	68
* *No	13	32
Radiation therapy		
* *Preoperative	1	2
Intraoperative/postoperative	15	37
* *Both (preoperative and postoperative)	3	7
* *None	22	54
Chemotherapy		
* *Neoadjuvant	5	12
* *Adjuvant	2	5
* *Both	1	2
* *None	33	81

**Table 3:** Univariate analysis of prognostic factors for overall survival in 41 patients with primary retroperitoneal sarcoma.

Variable	*N*	5-year survival (%)	*P*-value
Sex			0.89
* *Female	18	55	
* *Male	23	48	
Age			0.14
* * <55 years	16	76	
* * >55 years	25	33	
Duration of symptoms			0.53
* * <6 month	25	52	
* * >6 months	11	0	
* *Unknown	5	50	
Histological subtype			0.94
* *Liposarcoma	22	77	
* *Leiomyosarcoma	7	60	
* *MFH	4	33	
* *Other	8	0	
Tumor size			0.26
* * <5 cm	2	100	
* *5–10 cm	4	100	
* *10–20 cm	18	21	
* * >20 cm	17	60	
Tumor grade			**0.05**
* *Low	17	100	
* *High	24	27	
Resection			0.39
* *R0/R1	38	42	
* *R2	3	50	
Resection high-grade tumor only			**0.03**
* *R0/R1	22	74	
* *R2	2	44	
Radiation therapy			0.40
* *Yes	19	53	
* *No	22	63	
Chemotherapy			0.57
* *Neo-adjuvant	5	30	
* *Adjuvant	2	—	
* *Both	1	—	
* *None	33	43	

Multivariate analysis significant for grade (*P* = .006).

**Table 4:** Univariate analysis of prognostic factors for disease-free survival in 38 patients who underwent complete resection of their primary retroperitoneal sarcoma.

Variable	*N*	5-year survival (%)	*P*-value
Sex			0.69
* *Female	16	37	
* *Male	22	12	
Age			0.87
* * <55 years	15	35	
* * >55 years	23	0	
Duration of symptoms			**0.02**
* * <6 month	22	26	
* * >6 months	11	0	
* *Unknown	5	33	
Histological subtype			0.91
* *Liposarcoma	20	50	
* *Leiomyosarcoma	6	40	
* *MFH	4	33	
* *Other	8	0	
Tumor size			0.16
* * <5 cm	2	50	
* *5–10 cm	4	0	
* *10–20 cm	17	23	
* * >20 cm	15	—	
Tumor grade			0.84
* *low	16	0	
* *High	22	28	
Radiation therapy			0.29
* *Yes	19	0	
* *No	19	26	
Chemotherapy			0.87
* *Neo-adjuvant	5	—	
* *Adjuvant	2	33	
* *Both	1	0	
* *None	30	12	
